# Exploring the Relationship Between Day-to-Day Care and Caregiver Vigilance

**DOI:** 10.1093/geroni/igaa057.2191

**Published:** 2020-12-16

**Authors:** Brandy Renee McCann, Karen Roberto, Rosemary Blieszner, Tina Savla, Emily Hoyt, Aubrey Knight

**Affiliations:** 1 Virginia Tech, Blacksburg, Virginia, United States; 2 Virginia Polytechnic Institute and State University, Blacksburg, Virginia, United States; 3 Virginia Tech Carilion School of Medicine, Roanoke, Virginia, United States

## Abstract

A demanding aspect of caregiving for a relative with dementia is need for constant vigilance of their behavior and well-being. Vigilance is associated with higher quality of care, but can take a toll on caregivers who have few opportunities for respite. Home- and community-based services have the potential to offer caregivers relief from constant vigilance. Using in-depth interviews with 30 rural caregivers, we found that service use did not necessarily provide relief from constant vigilance. Caregivers typically needed to monitor aspects of home- and community-based services and care facilities such as scheduling and quality of care from CNAs. In contrast, some caregivers found respite from constant vigilance when they used formal services—typically in extreme situations such as when a husband with dementia became violent and moved temporarily to a long-term care facility. Findings connect caregiver needs and concerns related to vigilance with availability, quality, and use of formal services.

